# An Update on Anti-CD137 Antibodies in Immunotherapies for Cancer

**DOI:** 10.3390/ijms20081822

**Published:** 2019-04-12

**Authors:** Dinh-Toi Chu, Nguyen Duy Bac, Khanh-Hoang Nguyen, Nguyen Le Bao Tien, Vo Van Thanh, Vu Thi Nga, Vo Truong Nhu Ngoc, Duong Thi Anh Dao, Le Ngoc Hoan, Nguyen Phuc Hung, Nguyen Thi Trung Thu, Van-Huy Pham, Le Nguyen Vu, Thuy Anh Vu Pham, Deepak B. Thimiri Govinda Raj

**Affiliations:** 1 Faculty of Biology, Hanoi National University of Education, Hanoi 100000, Vietnam; daodangduc@gmail.com (D.T.A.D.); lengochoan@gmail.com (L.N.H.); hungnp@hnue.edu.vn (N.P.H.); trungthuhnue@gmail.com (N.T.T.T.); 2 School of Odonto Stomatology, Hanoi Medical University, Hanoi 100000, Vietnam; votruongnhungoc@gmail.com; 3 Institute of Cancer Research, Oslo University Hospital, 0372 Oslo, Norway; deepak.balaji@bitsaa.org; 4 Department of Education and Training, Vietnam Military Medical University, Hanoi 100000, Vietnam; bac_hvqy@yahoo.com; 5 National Food Institute, Technical University of Denmark, 2800 Kgs. Lyngby, Denmark; khng@food.dtu.dk; 6 Institute of Orthopaedics and Trauma Surgery, Viet Duc Hospital, Hanoi 100000, Vietnam; bstiencsvd@gmail.com (N.L.B.T.); thanhvo@hmu.edu.vn (V.V.T.); 7 Institute for Research and Development, Duy Tan University, 03 Quang Trung, Danang 550000, Vietnam; 8 AI Lab, Faculty of Information Technology, Ton Duc Thang University, Ho Chi Minh City 700000, Vietnam; 9 Organ Transplantation Center, Viet Duc Hospital, Hanoi 100000, Vietnam; nguyenvu.urologue@gmail.com; 10 Faculty of Odonto-Stomatology, University of Medicine and Pharmacy, Ho Chi Minh City 700000, Vietnam; pavthuy@ump.edu.vn

**Keywords:** CD137/4-1BB, CD137L/4-1BBL, anti-CD137 antibodies, cancer immunotherapies, anti-CD137 antibody therapy

## Abstract

The selective expression of CD137 on cells of the immune system (e.g., T and DC cells) and oncogenic cells in several types of cancer leads this molecule to be an attractive target to discover cancer immunotherapy. Therefore, specific antibodies against CD137 are being studied and developed aiming to activate and enhance anti-cancer immune responses as well as suppress oncogenic cells. Accumulating evidence suggests that anti-CD137 antibodies can be used separately to prevent tumor in some cases, while in other cases, these antibodies need to be co-administered with other antibodies or drugs/vaccines/regents for a better performance. Thus, in this work, we aim to update and discuss current knowledge about anti-cancer effects of anti-CD137 antibodies as mono- and combined-immunotherapies.

## 1. Introduction

Cancer immunotherapy is the type of treatment that restores or boosts the natural defence against tumor by the human immune system. Such therapy usually targets specific biological molecules on cancer cells’ surface such as tumor-associated antigens (TAAs). Anti-tumor activities are possibly achieved by directing the host immune system toward TAAs, which consequently establishes or induces adaptive immune responses against cancer cells. Over the past few decades, cancer treatment using monoclonal antibodies (mAbs) has achieved great success and many of them have been approved for cancer treatment or clinical trials. Some great examples of approved mAbs are alemtuzumab (anti-CD52), ipilimumab (anti-CTLA4), nivolumab (anti-PD1), rituximab (anti-CD20), urelumab, and utomilumab or PF-05082566 (anti-CD137 antibody). CD137 (4-1BB/TNFRSF9) was identified in 1989 as an inducible gene that was expressed on antigen-primed T cells but not on resting ones [1]. In addition, it is known to be expressed in dendritic (DCs), natural killer cells (NKs) [2], activated CD4^+^ and CD8^+^ T lymphocytes, eosinophils, natural killer T cells (NKTs), and mast cells [1,3,4,5], but myeloid-derived suppressor cells (MDSCs) were investigated to not express this molecule on their surfaces [6]. The anti-CD137 antibodies show great potential in anti-cancer activities due to its ability to activate cytotoxic T cells and to increase the production of interferon gamma (IFN-γ) [7,8]. Here, we will update and discuss the recent findings regarding clinical development of anti-CD137 antibodies (Table 1) as a cancer immunotherapy, either as monotherapy or combined treatment with other mAbs and/or reagents.

## 2. TNF Family Members and CD137 (L) Biology

The tumor necrosis factor receptor superfamily (TNFRSF) is a protein superfamily consists of 29 members [9] and plays important roles in human immune system [10]. This family of molecules is classified into two groups: death receptors (8 members) and activating receptors. All of them contain an intracellular signaling pathway activation domain and an extracellular receptor site. These receptor sites can be activated by binding to corresponding ligands of the tumor necrosis factor superfamily (TNFSF) [11,12]. It has been demonstrated that the ligand-receptor (e.g., CD40-CD40L, CD27-CD70 or OX40-OX40L) signaling pathways of TNFSF-TNFRSF can modulate many important processes in the body such as cell development and death of cells or induction of cytokines and chemokines [11]. Growing evidence also suggests that the TNFRSF and TNFSF members are involved in inflammatory and pathology in several diseases including cancer [13,14,15]. Consequently, immunotherapeutic developments targeting the ligand-receptor interactions of TNFSF and TNFRSF are highly potential for treatment of cancer.

In immunotherapy, an effective immune response requires two types of biological signal to fully activate T cells and other immune cells. The first signal, namely antigen-specific signal, is generated by the interaction of lymphocyte receptor with specific peptides bound to major histocompatibility complexes (MHC) molecules on antigen-presenting cells (APCs). Co-stimulation signal is the second signal and antigen-nonspecific. This type of signal is provided through the interaction between T cells and co-stimulatory molecules expressed on APCs [16]. CD137 is a co-stimulatory molecule belonging to the TNFRSF. It was discovered in the late 80s during T-cell-factor-screening on mouse helper and cytotoxic cells stimulated by concanavalin A [1]. In 1993, the human homologue of murine CD137 was identified [17]. CD137 can be activated by binding to its ligand (CD137L; 4-1BBL). The human CD137L was first isolated in 1994 using direct expression cloning from an activated CD4^+^ T lymphocyte population [18]. The lymphocytes were stimulated by immobilized CD3 monoclonal antibodies which bind to a fusion protein consisted of the extracellular portion of human CD137 coupled to the Fc region of human immunoglobin G1 [18]. The CD137L is expressed mostly on DCs, B cells or macrophages [19].

In mice, CD137 ligation results in recruitment of TNFR factors (TRAF1 and TRAF2) consequently leads to the activation of the nuclear factor kappa-light-chain-enhancer of activated B cells (NF-κB), Jun amino-terminal kinases/Stress-activated protein kinases (JNK/SAPK) and p38 mitogen-activated protein kinases (p38 MAPK) pathways [20]. This generates co-stimulatory signals to induce the activities of both CD4^+^ and CD8^+^ T lymphocytes [20], however, the proliferation of CD8^+^ T cells is more favourable [8]. The activation of CD4^+^ T lymphocytes via p38 MAPK is also essential for development of T helper (Th) 1 and 2 cells as well as modulation of Th1 effector cells [20]. It was later proved that murine CD137 ligation by an agonist monoclonal antibody provided a long term survival signal to CD8^+^ T lymphocytes and thus inhibited activation-induced cell death (AICD) [21,22]. The CD137/CD137L pathway also affects non-T cells in the immune system. The CD137/CD137L co-stimulation involves in controlling of monocyte numbers by amplification of monocyte-mediated proliferation of invariant NKT cells [23]. CD137 and/or CD137L agonists stimulate production of several inflammatory cytokines such as IL-6, TNF-α, and MCP-1 in adipocytes and macrophages [24]. Crosslinking of CD137 on B cells generates co-stimulatory signal to activate and induce proliferation of B cells [25]. Anti-CD137 antibody therapy has been shown to severely deplete CD4, B cells and NK cells. Due to the capacity to stimulate both innate and adaptive leukocytes of CD137/CD137L, several research papers have targeted this signaling pathway using agonistic anti-CD137 antibodies as a potential therapeutic treatment of cancer.

## 3. Agonistic Anti-CD137 Antibody Monotherapy (Anti-CD137 mAb) for Cancer Treatment

Agonistic anti-CD137 mAb has been demonstrated to induce and improve anti-cancer immunity in several models of cancer [26,27,28,29,30,31,32,33,34,35,36]. The mechanisms underlying tumor regression by this antibody are its effects on pivotal immune cells regulating body’s immune responses to cancer cells. Anti-CD137 antibody stimulates and activates effector T lymphocytes (e.g., stimulating CD8 T lymphocytes to produce INFγ), NKTs, and APCs (e.g., macrophages) [26,28,30,32,33,35,37,38] to kill oncogenic cells selectively. Under the stimulation of anti-CD137 mAb the number and activities of tumor antigen-specific memory T cells are also increased resulting in prolongation of immune responses against cancer cells [27]. Several TNF receptors including CD137 function mainly as costimulatory factors for T cell activation. In addition, costimulation of CD137 activates Akt to promote cell cycle through regulation of cyclins and cyclin-dependent kinases. However, the roles of this antibody on NKs are still controversial: this antibody activates mouse NK cells while it can either activate or inhibit human NK cells depending on the setting or models [39]. On the other hand, this antibody inhibits the proliferation and activity of immune-suppressing cells such as MDSCs and regulatory T cells [28,35,38,40]. It was also reported that anti CD137 antibody blocked IL-9 production to inhibit T regulatory cells [40].

The effects of anti-CD137 antibody on tumor immunity are based on co-stimulation through the CD137 receptor on immune cells, oncogenic cells, and other cells in tumor microenvironment. Thus the expression of CD137 on both target cells and host cells is required, and genetic CD137 ablation on either tumor cells or host cells (Antigen-specific memory T cells and cells from hematopoietic and nonhematopoietic origins) eliminates the antitumor effect of this antibody [27]. Specific presence of CD137 on the surface of tumor-associated endothelial cells augments immigration of activated T cells into the malignant tissue [29]. Moreover, anti-CD137 mAb-elicited rejection of lymphomas needs cytolytic coaction of both perforin-granzyme and FAS-L mechanisms [30].

Agonistic anti-CD137 antibody enhances anti-cancer immunity to protect mice from liver tumor [26]. This outcome is due to an active effect of cytotoxic T cells, NK cells, and macrophages by this antibody [26]. In addition, the antibody also limits the infiltration of cells suppressing anti-tumor immunity such as MDSC and regulatory T lymphocytes [26]. Furthermore, in a mouse model of malignant melanoma, anti-CD137 antibody prevents recurrence and metastases of cancer after removal of primary tumors by expanding antigen-specific memory T lymphocytes [27]. The overexpression of CD137 in bulk tumor specimens of cancer patients attracted attention to test anti-cancer action of anti-CD137 mAb in lymphoma models [28]. In mice, anti-CD137 mAb effectively prevents lymphoma as indicated by inducing the regression of tumor size and increase of survival percentage, since it increases the functions of NKs and CD8 lymphocytes, but suppresses regulatory T cells [28]. Notably, the anti-lymphoma immunity simulated by this antibody is long lasting [28]. These anti-tumor activities of anti-CD137 mAb have also observed in the murine models of lung (M109) and breast (EMT6) cancer [31]. treatment with an anti-CD137 antibody (BMS-469492) modestly inhibits growth retardation of M109 tumors, but strikingly prevents the development of EMT6 tumors, especially in a combination with irradiation [31].

Accumulating evidence in animal cancer models shows that anti-CD137 antibodies have a therapeutic potential to prevent cancer in human being. In a pre-clinical study, a human IgG2 conjugated to extracellular region of CD137, namely PF-05082566, effectively prevents development in a xenograft model of human cancer [41]. This anti CD137 antibody activates NF-κB, induces downstream cytokine production in both human and monkey leukocytes, and promotes CD8^+^ T cell proliferation in monkey [41]. Anti-CD137 mAb has been considered for clinical trials in patients with advanced cancer [33,37,42,43], but its anti-cancer ability is supposed to be various due to inter-species differences in binding sites of anti-CD137 antibody on CD137 receptor. Specifically, its binding interaction is limited to cysteine-rich domain (CRD) and it differed between the mouse and human. Mouse CD137L mainly combined with CRDII and human CD137L mainly combined with CRDIII [42]. Briefly, Human and mouse CD137 CRD has four CRDs (CRDI, II, III and IV). CRDI and CRDIV regions both contain only four cysteines that cannot form the typical CRD structure while their CRDII and CRDIII both contain six cysteines that can form the typical CRD structure.

Currently, two ongoing clinical trials have been being set for two anti-CD137 mAbs namely urelumab (BMS-663513) and utomilumab (PF-05082566) [44]. They are fully human IgG4 (urelumab) or IgG2s (utomilumab) mAbs which were developed by Bristol-Myers Squibbb or Pfizer, respectively. Urelumab showed a promising cancer treatment potential in a preclinical study [45]. However, the liver toxicity of the antibody has emerged shortly afterward which caused urelumab development program was put on hold during the period December 2008 to February 2012 [46]. This toxicity was mainly due to S100A4 protein secreted by tumor and stromal cells [47]. Recent studies suggested that this antibody product is safe at a dose of 8 mg or 0.1 mg/kg per patient for every 3 weeks [46,48]. In comparison with urelumab, utomilumab showed better safety profile as no dose-limiting toxicities (DLTs) or elevations of liver enzymes observed in a 27 cancer patient cohort [49]. Recently, the outcomes from a phase I trial of utomulumab monotherapy in 55 advanced cancer patients were emerged [50]. Data from the trial supported a well-tolerated safety profile of utomilumab where no DLTs were observed at the dose levels of up to 10 mg/kg for every 4 weeks. Additionally, only less than 10% of patients experienced treatment-related adverse events, most of them were grade 1 or grade 2 events such as dizziness or rash. The rates of overall objective response and the best overall response in patients with solid tumors were 3.8 and 24.5%, respectively, including 1 complete response [50]. In clinical setting, toxicity of urelumab or utomilumab is completely understood and this could be due to the mechanistic differences in their agonist activity or CD137 binding properties [51]. Comparison between utomilumab- and urelumab-bound receptor structures showed that the mAbs have dramatically different binding sites, in terms of epitope and relative orientation of the mAbs. Utomilumab binds along the side of CD137, making contact at the junction between CRDs III and IV. In contrast, urelumab binds in the N-terminus of the CD137 receptor on CRD I. Clinical efficacy results with lose-dose urelumab monotherapy were largely ineffective and there has been limited clinical activity of urelumab at the tolerated dose. While It has been reported that Utomilumab has a better safety profile, but is a less potent CD137 agonist relative to urelumab [52]. Due to its better safety profile, Utomilumab is preferred mAbs for the combination therapy compared to urelumab.

In hematological tumors, mono-immunotherapy of anti-CD137 antibody could regulate haematological malignancies in some models [53,54]. In a mouse model of multiple myeloma, this antibody could rescue 40–50% of mice in the treated group compared to control one [54]. Anti-CD137 mAb also suppressed the growth of acute myeloid leukemia (AML) in 50% experimented animals [53]. These anti-hematological tumor outcomes of anti-CD137 mAb were due to an increase in the generation and IFN-γ production of CD8 T cells [54].

## 4. Combined Therapy of Anti-CD137 mAb and Other Antibodies for Cancer Treatment

In several models of cancer, a specific antibody against CD137 receptor has synergistic effects with other antibodies on inhibiting tumors [33,34,55,56,57,58,59,60,61,62,63]. In these combined therapies, the antibodies support each other to achieve higher immune responses against cancer compared to single treatments.

Regulatory barriers such as regulatory T lymphocytes, indoleamine-2,3-dioxygenase DCs or T-cell immunoglobulin and mucin domain 3 (TIM-3) can suppress immune responses by the immune system toward oncogenic antigens. This explains why the deletion of regulatory barriers becomes a target to improve immune response towards cancer in an anti CD137 mAb trial [56,64]. Cell division caused by oncogenic stimulations without growth factor such as mutation is developmental origin of cancer. Turning off uncontrolled growth by blockage of epidermal growth factor receptor (EGFR) using a human-mouse chimeric IgG1 mAb (Cetuximab) results in regression of colorectal carcinoma, and head and neck cancer in patients [65]. This effect of Cetuximab is strengthened by co-administration with anti-CD137 mAb [66]. Cetuximab upregulates CD137 expression on NK cells in patients, the expression of this costimulatory molecule elicits NK cell and memory T cell activation [66]. Anti-CD137 mAb, in its turn, improves NK cell degranulation and cytotoxicity by activating CD137 receptor to strengthen anti-cancer immunity [66]. S100A4 is a calcium-binding protein and has a critical role in inflammatory responses and liver pathogenesis [67,68,69]. As discussed above, the anti-CD137 mAb can induce liver toxicity mainly through S100A4 secretion. A combined immunotherapy of anti-CD137 and anti-S100A4 mAbs showed stronger antitumor responses compared to anti-CD137 monotherapy and also significantly reduced liver pathology in mouse models [47]. Yonezawa A., et al. has elaborated the current status of clinical trials of anti-CD137 agonistic mAbs in Table 3 of their report [70].

Programmed death-1 (PD1) provides inhibitory signals which maintain T lymphocytes to functionally silence against cognate antigens; therefore interference of PD1 by its specific antibody results in activation of T cells, and subsequently enhances anti-tumor immunity under a combined treatment of PD-1 blockage and anti-CD137 antibody [55,60]. Treatment of lung cancer beyond day +17 in murine models by either anti PD1 or anti CD137 mAbs was not effective while a combination of the two antibodies showed complete tumor rejection [52]. Superior efficacy enhancement was obtained by sequential administration of anti-CD137 and anti-PD1 mAbs rather than either monotherapy for B-cell lymphoma in mice and the synergistic therapy was dependent on Fc receptors [70]. The primary mechanism behind the anticancer activity of mAbs is Abs-dependent cell-mediated cytotoxicity (ADCC). ADCC involves NK cells which bears an Fc receptor (CD16) binds to the antibody-targeted tumor cell and facilitate tumor cell lysis. The same observation was witnessed in B16 melanoma-bearing mice where an anti-CD137/anti-PD combination reduced cancer development more effectively than a single mAb treatment with anti-CD137, -PD1, -CTLA-4 or -CD4 did [71].

In pre-clinical trials, the combined immune therapies of anti-CD137 mAb with anti-PD1, -CTLA-4 or -CD4 antibodies have brought positive outcomes for some cancer medication [34,55,56,57,58,62,64], but not for some other cancers such as a combination of anti-CD137 and anti-PD1 antibodies in a mouse model of B-cell lymphoma [72]. Blockade of PD1 protein by a specific antibody supports anti-CD137 mAb in treating ovarian and lung cancer in murine models [55,62]. A mixed therapy of anti-CD137 and anti-PD1 mAbs increases the survival rate of tumor-bearing mice by inducing a systemic response of CD8 lymphocytes and immunological memory [55]. However, this combination cannot prevent established subcutaneous AT-3 tumor in a mouse model without radiotherapy [73]. A combined therapy of anti-CD137 and anti-CD40 antibodies leads to tumor regression and lifetime prolongation of tumor-bearing mice with colon cancer and lymphoma, but this combination has modest effects on preventing multiple lymphomas [57]. CD4 T cell depletion or TIM-3 blockade by specific antibodies brings more benefits to the anti-cancer action of anti-CD137 mAb [56]. Anti-CD4 antibody removes regulatory lymphocytes and indoleamine-2,3-dioxygenase DCs to enhance the infiltration of immune cells into tumor tissues. Therefore, a mixed injection of anti-CD4 and andti-CD137 antibodies results in significant regression of cancer in melanoma-bearing mice [56]. Whereas, combined anti-TIM-3 treatment and CD137 activation provide stronger immune responses against ID8 ovarian cancer not only by inhibiting regulatory T cells and activating CD8 lymphocytes but also by promoting memory CD4 lymphocytes and decreasing MDSC in the immune system [64].

Blockage of interaction between cytotoxic T-lymphocyte-associated protein 4 (CTLA-4) and CLTA-4 ligands by an antibody enhances overall immune responses [59], which contributes to significant regression of colon cancer and melanoma by a mixed injection of anti-CD137 and -CTLA-4 antibodies [34,38,58,61]. This combination strategy improves anti-cancer immunity through the antigen-specific enhancement of T CD8 cell activities and immune memory, but suppresses autoimmunity via increased function of regulatory T lymphocytes [58]. The conjugation of anti-CTLA-4 and anti CD137 antibodies also prevented significantly the cancer development in mouse model of B16 melanomas when this therapy was combined with a B16-Flt3- ligand (FVAX) vaccine, this outcome was due to the increasing in T lymphocyte infiltration and proliferation as well as enhancement of cytokine production [38]. Further investigations show that the combined immunotherapy of anti-CTLA-4 and-CD137 antibodies only demonstrate efficacy when tumor-specific T-cell repertoire has not been exhausted [74].

Anti-CD137 mAb also shows the benefits in the triple combination of antibodies in cancer treatment [60,63]. Anti-PD1 with -CTLA-4 antibodies counter immunosuppressive Th2 to support anti CD137 mAb in regression of established tumor [60], the anti-tumor activities of these antibodies are enhanced by radiotherapy [75]. In cooperation with anti-OX40 and anti-PD-L1 antibodies, the anti CD137 antibody prevents hepatocellular carcinoma in mice by increasing effector T lymphocytes [63]. In a mouse model of CT26 tumor, anti-CD137 mAb has synergistic activities with PD-L1/B7-H1 blockage by a specific antibody. This anti-tumor effect of the antibody combination is increased by the HIF-1α hypoxia response in the T cells in tumor microenvironment [36]. Notably, a low intratumoral dose of anti-CD137 mono antibody brings an immune response against cancer without any side effects (e.g., liver inflammation) [36]. Recently, a novel low dose (10 μg each) triple combination of anti-CD137, -CTLA4 and -OX40 mAbs was effective in the treatment of cancer in the large intestine and B-cell lymphoma in mouse models [76]. On the contrary, recent research reported diminished therapeutic potential of anti-CD137 mAb by anti-PD1 mAb to control B-cell lymphoma in Eμ-myc transgenic mice [72].

In a xenotransplant model of a breast tumor, anti-CD137 mono antibody increases anti-cancer effects of Trastuzumab, a mAb targeting the human epidermal growth factor receptor 2 (HER2 or HER-2/neu) [77]. In this combination, Trastuzumab stimulates NK cell activity and enhances expression of CD137 in these cells both in vitro and in vivo, then anti-CD137 mono antibody elevates the trastuzumab-regulated NK cell cytotoxicity and cytokine release to kill tumor cells [77]. Anti-CD137 mAb also supports trastuzumab in the treatment of breast carcinomas in mice [78]. A phase Ib clinical trial of utomilumab in a combination with pembrolizumab (an anti-PD1 antibody) for 23 subjects having advanced solid tumors reported 26.1% response (complete or partial) and no DLTs; elevated levels of effector/memory CD8^+^ lymphocytes were observed in responded patients [79]. These findings encourage us to develop more clinical trials for cancer treatment by combination therapies of anti CD137 mAb with other antibodies.

## 5. Other Combinations with Anti-CD137 mAb for Cancer Treatment

Mono antibody against the CD137 receptor has negligible or no anti-cancer effect on poorly immunogenic tumor cells (weak tumor antigens) such as B16-F10 melanomas, 3-methylcholanthrene–induced fibrosarcomas, and TC-1 lung carcinomas [38,55,57,58,80,81,82,83,84,85]. Therefore, the combination of the anti-CD137 antibodies with other reagents is necessary.

An adenovirus carrying mIL-12 (ADV/IL-12) has been developed and proved to support anti-CD137 mAb fighting subcutaneous and lung metastatic melanoma caused by B16-F10 tumor cells [80]. IL-12 induced by this recombinant virus stimulates NK cells to stimulate the immune system, which results in recruitment of CD8 T lymphocytes to tumor microenvironment, while antibody against CD137 increases the function of these recruited T cells [80]. Systemic injection of this antibody enhances the eradicating efficacy of IL-12-secreting DC therapy against colon cancer [81]. IL-12 also supports anti-CD137 mAb on treating liver cancer [86]; a combined therapy of ADV/IL-12 and a specific antibody against CD137 rejects effectively metastatic bowel cancer developed in liver by increasing the functions of NK and CD8 T lymphocytes. A combination of SFV-IL-12 (a semliki Forest virus encoding IL-12) and anti-CD137 antibody provides powerful synergistic effects to prevent melanoma and lung carcinoma in mice [83]. Intratumoral injection of this IF-12 producing-virus induces high level of CD137 on infiltrated CD8 T lymphocytes in tumor area, which provides more selective targets for anti-cancer activity of the mAb [83].

This antibody augments the activities of granulocyte-macrophage colony-stimulating factor (GM-CSF) secreting cells to treat cancer [87], the combined treatment of GM-CSF secreting cell-therapy and anti-CD137 mAb results in significant abrogation of established B16 melanoma through the maintenance of tumor-infiltrating CD8 T lymphocyte activation, and expansion of memory lymphocytes in tumor microenvironment [87]. Injection of anti-CD137 mAb fixes the limitation of 5-fluorouracil (5-FU) in inhibiting renal carcinoma [88]. Combined treatment of anti-CD137 mAb and 5-FU effectively blocks more than 70% of tumor-bearing mice by increasing infiltration of effector lymphocytes [88].

The same result is obtained by a combined treatment of anti-CD137 mAb with tumor lysate-pulsed dendritic cell (TP-DC) vaccine in mouse models of established lung carcinoma and subcutaneous cancer [82]. TP-DC vaccination upregulates the expression of CD137 on NKs within vaccine-primed lymph nodes (VPLNs), anti-CD137 mAb stimulates the anti-cancer activity of type 1 T effector cells in VPLNs and spleen as indicated by a high level of interferon γ in these locations [82]. Another example of vaccine therapy application to anti-cancer immunity of anti-CD137 mAb is the engineered strain of oncolytic vaccinia virus (Vvdd): cooperation of the antibody with Vvdd has successfully inhibited established subcutaneous tumor, while either Vvdd or anti-CD137 antibody treatment alone had slight effects on these tumor models [89]. An administration of anti-HVEM scFv-expressing cancer vaccine (α-HVEM scFv vaccine) activates an immune response preventing mastocytoma and lymphoma in mouse models through the development and activity of CD8 lymphocytes and memory T lymphocytes [90]. These anti-tumor activities of α-HVEM scFv vaccine are significantly increased by an injection of anti CD137 antibody [90].

Anti-hematological tumor effects of anti-CD137 Ab are enhanced by other therapies [53,72,91]. A combination of this antibody with alpha-galactosylceramide-loaded cancer cell vaccination led to increasing a protective rate in a mouse model of acute myeloid leukemia (ALM) by 100% compared to anti-CD137 treatment separately [53]. The similar results were found when anti-CD137 antibody was administered with a vaccine targeting NKT cells, the combination resulted in 50–70% long-term survival in a murine model of B-cell lymphoma [72,91]. Both clinical trials of anti-CD137 antibodies, utomilumab and urelumab, have shown promising outcomes when combined with other immunotherapies to treat lymphoma patients [52]. The mechanisms underlying elevated effects against cancer of these combinations were the enhancement in CD8 T cell activation and IFN-γ production [53], and the increase in CD8 T cell generation and expansion [91].

In a cooperation with an oligodeoxynucleotide (CpG1826), anti-CD137 antibody effectively treats mesenteric kidney tumor; this activity requires the action of CD8 lymphocytes and performs better with the intravenous route of CpG delivery [84]. In order to block pathological angiogenesis of cancer, hybrids of DCs and syngeneic endothelial cells (EC) are generated to favour anti-CD137 mAb fighting against tumors [85]. DC-EC hybrids elicit EC-specific CD8 T and CD4 lymphocyte activities those self-antigen–selective immune responses which inhibit tumor angiogenesis to strengthen the effects of anti-CD137 antibodies on preventing B16.F10 melanoma as well as MC38 colon adenocarcinoma [85]. A co-stimulation triggered by anti-CD137 antibody elevates the proliferation and action of effector memory CD8 lymphocytes infiltrating into cancer microenvironment therefore potentially improves the tumor prevention of an adaptive T-cell therapy (ACT) [32].

There are evidence that innate immunity is also involved in anti-cancer effects of anti-CD137 antibody in some combinations, especially for treating hematological tumor [52,92,93]. It is proved that the action of this antibody against cancer cells is NK cell-dependent, particularly through the proliferation and IFN-γ secretion, and cytotoxic action of these innate lymphoid cells [92,93].

Nearly half of melanomas expresses proto-oncogene B-Raf (BRAF) mutant, blockage of BRAF by its inhibitor, PLX4720, down-regulates tumor Ccl2 genes but increases the tumor infiltration and activates of CD8 lymphocytes and NK cells [94]. Therefore, treatment of mice bearing mesenteric melanomas by a mix of PLX4720 with anti-CD137 stimulates a better anti-cancer activity [94]. The combined treatment of anti-CD137 antibody and interleukin 2 has shown potential to prevent tumor in vivo, but this is limited due to inflammatory toxicities [95]. A new method to deliver this immune combination to tumor microenvironment, named the local nanoparticle-anchored delivery, has brought significant anti-melanoma outcomes in a mouse model with modest systemic toxicities [95]. Similarly, anchoring IL-2 and antibody against CD137 onto liposome surface provides the same anti-tumor activity as non-anchored drugs combined treatment without systemic toxicity in several cancer models [96].

## 6. Prospects and Future Directions

Immunotherapies for checkpoint blockade have shown as effective methods to treat several types of cancer, by they also brought about remarkable changes in clinical practice in a wide variety of tumor indications. The most famous antibodies used as cancer immunotherapies, which are intensively studied, are anti-CTLA4 and -PD-1 antibodies [97]. One of the significant outcomes of these immunotherapies in cancer treatment is the medicine Nobel Prize 2018 which was given to Profs. James Allison and Tasuku Honjo who are pioneers on discovering the “checkpoint” proteins, CTLA-4 and PD-1 [98]. The cooperative anticancer effects of anti-CD137 with anti-CTLA4 or/and -PD-1 antibodies in several cancer types lead to promising directions in applying anti-CD137 antibody in the immune checkpoint therapies to advance cancer treatment. Thus, more investigations on the combination of anti-CD137 and other antibodies and/or other reagents to treat cancer and on underlying mechanisms of their anti-cancer effects are needed. Furthermore, we also need more studies on the changes in application of immune checkpoint inhibitors to treat different cancer stages and various types of tumor.

## 7. Conclusions

**Figure 1:**
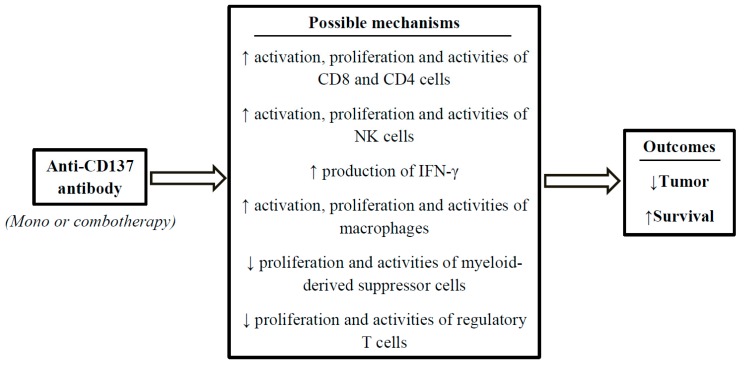
Schematic illustration of mechanisms underlying the anti-cancer effects of anti-CD137 antibodies in immunotherapies. Current evidence suggests that in mono or combotherapies for cancer, anti-CD137 antibodies increase anti-tumor immune response because they can activate/regulate immune subsets in the tumor microenvironment such as these antibodies increase (↑) the activation, proliferation and activities of CD8 T cells, CD4 T cells, natural killer (NK) cells and macrophages; increase IFN-γ production, but they inhibit (↓) the proliferation and functions of myeloid-derived suppressor cells and regulatory T cells.

Both basic and clinical studies in cells and animal models of cancer have shown that the anti-CD137 antibody is a potential cancer immunotherapy (Table 2 and Figure 1). However, the effects of antibodies against CD137 on tumor are various depending on types and developmental stages of cancer as well as the immunotherapies that have been used. Additionally, the hepatoxicity of the anti-CD137 mAb monotherapy is also an obstacle to developing anti-CD137 drugs. Combination therapies of anti-CD137 with other antibodies or other reagents have shown great potentials of anti-tumor activities and minimized the possibility of systemic toxicities (Table 3 and Table 4, and Figure 1). Alternative drug administration such as intratumoral injection or local nanoparticle-anchored antibodies also show advantages against systemic drug delivery. In conclusion, anti-CD137 antibodies have the therapeutic potential for cancer treatment but more clinical development is required to fully unlock the application of this antibody.

## Figures and Tables

**Table 1:** The list of anti-CD137 antibodies, their different properties and binding sites.

Name (Brand Name)	Agonist or Antagonist	Properties	Binding Sites
Urelumab (BMS-663513)	Agonist	Human IgG4 mAb	CRD I
Utomilumab (PF-05082566)	Agonist	Humanized IgG2 mAb	CRDs III and IV

**Table 2:** The effect of mono anti-CD137 therapy on tumor immunity.

Therapies	Cancer Models	Effects on Cancer Immunity	Ref
α-CD137 mAb	Mouse model of hepatocellular carcinoma	↓tumor, ↓MDSC, ↓regulatory T cells (Treg), ↑cytotoxic T lymphocytes (CTLs), ↑NK cells, ↑macrophages	[26]
α-CD137 mAb	Mouse model of lymphoma	↓tumor, ↑survival, ↑memory T cells, ↑NK cells, ↑CD8 T cells, ↓Treg	[28]
α-CD137 mAb	Mouse models of metastatic melanoma	↓tumor, ↓tumor recurrence and metastases, ↑survival, ↑antigen-specific memory T cells	[27]
α-CD137 mAb	Mouse model of thymomas	↓tumor, ↑survival	[30]
α-CD137 mAb	Mouse model of lung and breast carcinoma	↓tumor, ↑survival	[31]
α-CD137 mAb	Mouse model of B16 melanomas	↑survival, ↑CD8 and CD4 effector T cells, ↓Treg	[38]
α-CD137 mAb	Mouse mole of CT26 tumor	↓tumor, ↑survival	[36]
α-CD137 mAb (PF-05082566)	Mouse mole of prostate carcinoma	↓tumor, ↑CD8	[41]
Radiation + α-CD137 mAb	Mouse model of lung, breast carcinoma, and 4T1 cancer	↓tumor, ↑survival	[31,73,99]

mAb: Agonistic monoclonal antibodies; ↑: Increase; ↓: Inhibit; +: In a combination.

**Table 3:** The effect of combination between anti-CD137 and other antibodies on tumor immunity.

Therapies	Cancer Models	Effects on Cancer Immunity	Ref
α-PD-1 mAbs + α-CD137 mAb	Mouse models of ovarian and lung cancer	↑↑ survival, ↑CTLs, ↑memory T cells, ↓Treg	[55]
α-CTLA-4 Ab + α-CD137 mAb	Mouse models of colon cancer and melanoma	↓tumor, ↓Liver metastases, ↑CD8 T cells, ↑Treg	[58,74]
α-CTLA-4 Ab + α-CD137 mAb	Mouse model of B16 melanomas	↑survival, ↑CD8 and CD4 effector T cells, ↓Treg, ↑IFN-γ	[38]
α-PD-1 mAb + α-CTLA-4 Ab + α-CD137 mAb	Mouse models of ovarian carcinoma and subcutaneous melanoma	↓tumor, ↑survival, ↑IFN-γ and TNF-α producing CD4 and CD8 T cells, ↑mature CD cells, ↓Treg, ↓MDSC, ↓immunosuppressive Th2	[60]
α-B7-H1 Ab + α-CD137 mAb	Mouse moles of CT26 tumor	↓tumor, ↑survival, ↑CD137 on activated T lymphocytes	[36]
α-OX40 Ab + α-B7-H1 Ab + α-CD137 mAb	Mouse model of hepatocellular carcinoma	↓tumor, ↑survival, ↑CD8 and CD4 effector T cells	[63]
Radiation + α-PD-1 mAbs + α-CD137 mAb	Mouse model of AT-3 tumors	↓tumor, ↑survival, ↑CD137 on tumor-associated CD8 T cells	[73]
Trastuzumab + α-CD137 mAb	Xenotransplant model of human breast cancer	↓tumor, ↑survival, ↑CD137 on NK cells, ↑trastuzumab-mediated NK cell cytokine secretion and cytotoxicity	[77]
Trastuzumab + α-CD137 mAb	Mouse model of breast carcinomas	↓tumor, ↑survival	[78]
5-fluorouracil (5-FU) + CD137 mAb	Mouse model of renal carcinoma	↓tumor, ↑survival, ↑lymphocytes	[88]
α-CD40 mAb + α-CD137 mAb	Mouse models of colon cancer, lymphoma	↓tumor, ↑survival	[57]
α-CD4 mAb + α-CD137 mAb	Mouse model of melanoma	↓tumor, ↑survival, ↑CD8 T cells, ↑tumor-specific CTLs	[56]
α-TIM-3 Ab + α-CD137 mAb	Mouse model of ovarian cancer	↓tumor, ↑survival, ↑CD4 (memory) and CD8 (effector) T cells, ↓Treg and MDSC, ↑Th1 type, ↑IFN-γ	[64]
Cetuximab + α-CD137 mAb	Mouse models of head and neck cancer, colorectal cancer	↓tumor, ↑survival, ↑CD137 on NK cells, ↑NK cell degranulation and cytotoxicity	[66]
CpG1826 + α-CD137 mAb	Mouse model of kidney tumors	↓tumor, ↑survival	[84]

mAb: Agonistic monoclonal antibodies; Trastuzumab: a monoclonal antibody targeting human epidermal growth factor receptor 2; ↑: Increase; ↑↑: Strongly increase; ↓: Inhibit; +: In a combination.

**Table 4:** The effect of anti-CD137 and other combinations on tumor immunity.

Therapies	Cancer Models	Effects on Cancer Immunity	Ref
DC-EC hybrids + α-CD137 mAb	Mouse models of melanoma and colon adenocarcinoma	↓tumor, ↓tumor angiogenesis, ↑survival, ↑EC-specific T-cell responses	[85]
GM-CSF-secreting tumor cell + α-CD137 mAb	Mouse models of melanoma and colon carcinoma	↓tumor, ↑ survival, ↑CD8 T responses, ↑CD8 T cell infiltration, ↑memory responses	[87]
PLX4720 + α-CD137 mAb	Mouse model of metastatic melanoma	↓tumor, ↑ survival, ↓Ccl2, ↑CD8T cells, ↑NK cells	[94]
TP-DC vaccination + α-CD137 mAb	Mouse models of established pulmonary and subcutaneous tumor	↓tumor, ↑survival, ↓metastases, ↓local recurrence, ↑CD137 on NK cells, ↑antigen-reactive T cells	[82]
ADV/IL-12 + α-CD137 mAb	Mouse models of subcutaneous and lung metastatic melanoma	↓tumor, ↓pulmonary metastases, ↑ survival, ↑NK cells, ↑CD8 T cells,	[80]
ADV/IL-12 + α-CD137 mAb	Mouse model of liver cancer	↓tumor, ↑survival, ↑CD8 Tcell infiltration, ↑NK cell activation, ↑antigen-specific memory T cells	[86]
AdCMVIL-12-DCs + α-CD137 mAb	Mouse model of colon cancer	↓tumor, ↑survival, ↑IFN-γ	[81]
Vvdd + α-CD137 mAb	Mouse model of AT-3 tumors	↓tumor, ↑ survival, ↓pulmonary metastasis, ↑CD8 T cells, ↑NK cells	[89]
α-HVEM scFv vaccine + α-CD137 mAb	Mouse models of mastocytoma and lymphoma	↓tumor, ↑ survival, ↑CD8 T cells, ↑T cell memory	[90]
SFV-IL-12 + α-CD137 mAb	Mouse models of melanoma and lung carcinoma	↓tumor, ↑CD137 on tumor-infiltrating CD8 T cells, ↑CTLs, ↓Treg	[83]
IL-2Fc + α-CD137 mAb	Mouse models of melanoma	↓tumor, ↑survival, ↓systemic toxicity	[95]

mAb: Agonistic monoclonal antibodies; ADV/IL-12: A recombinant adenovirus expressing mIL-12; AdCMVIL-12-DCs: recombinant adenoviruses AdCMVmIL-12 injected dendritic cells; TP-DC vaccination: Tumor lysate-pulsed dendritic cell vaccination; Vvdd: engineered strain of oncolytic vaccinia virus; ↑: Increase; ↓: Inhibit; +: In a combination.

## References

[1] Kwon B.S., Weissman S.M. (1989). cDNA sequences of two inducible T-cell genes. Proc. Natl. Acad. Sci. USA.

[2] Vinay D.S., Kwon B.S. (2012). Immunotherapy of Cancer with 4-1BB. Mol. Cancer Ther..

[3] Vinay D., Kwon B. (2006). Immunotherapy Targeting 4-1BB and Its Ligand. Int. J. Hematol..

[4] Croft M. (2009). The role of TNF superfamily members in T-cell function and diseases. Nat. Rev. Immunol..

[5] Pollok K., Kim Y., Zhou Z., Hurtado J., Kim K., Pickard R., Kwon B. (1993). Inducible T cell antigen 4-1BB. Analysis of expression and function. J. Immunol..

[6] Dubrot J., Azpilikueta A., Alfaro C., Murillo O., Arina A., Berraondo P., Hervás-Stubbs S., Melero I. (2007). Absence of surface expression of CD137 (4-1BB) on Myeloid-derived suppressor cells. Inmunología.

[7] Melero I., Johnston J.V., Shufford W.W., Mittler R.S., Chen L. (1998). NK1.1 Cells Express 4-1BB (CDw137) Costimulatory Molecule and Are Required for Tumor Immunity Elicited by Anti-4-1BB Monoclonal Antibodies. Cell. Immunol..

[8] Shuford W.W., Klussman K., Tritchler D.D., Loo D.T., Chalupny J., Siadak A.W., Brown T.J., Emswiler J., Raecho H., Larsen C.P. (1997). 4-1BB Costimulatory Signals Preferentially Induce CD8+ T Cell Proliferation and Lead to the Amplification In Vivo of Cytotoxic T Cell Responses. J. Exp. Med..

[9] Collette Y., Gilles A., Pontarotti P., Olive D. (2003). A co-evolution perspective of the TNFSF and TNFRSF families in the immune system. Trends Immunol..

[10] Li J., Yin Q., Wu H. (2013). Structural basis of signal transduction in the TNF receptor superfamily. Adv. Immunol..

[11] Croft M., Duan W., Choi H., Eun S.Y., Madireddi S., Mehta A. (2012). TNF superfamily in inflammatory disease: Translating basic insights. Trends Immunol..

[12] Locksley R.M., Killeen N., Lenardo M.J. (2001). The TNF and TNF Receptor Superfamilies. Cell.

[13] Marín N.D., García L.F. (2017). The role of CD30 and CD153 (CD30L) in the anti-mycobacterial immune response. Tuberculosis.

[14] Pelekanou V., Notas G., Theodoropoulou K., Kampa M., Takos D., Alexaki V.-I., Radojicic J., Sofras F., Tsapis A., Stathopoulos E.N. (2011). Detection of The TNFSF Members BAFF, APRIL, TWEAK and Their Receptors in Normal Kidney and Renal Cell Carcinomas. Anal. Cell. Pathol..

[15] Lee C., Park J.-W., Suh J.H., Moon K.C. (2015). High expression of APRIL correlates with poor prognosis in clear cell renal cell carcinoma. Pathol. Res. Pract..

[16] Tabrizi M., Zhang D., Ganti V., Azadi G. (2018). Integrative Pharmacology: Advancing Development of Effective Immunotherapies. AAPS J..

[17] Schwarz H., Tuckwell J., Lotz M. (1993). A receptor induced by lymphocyte activation (ILA): A new member of the human nerve-growth-factor/tumor-necrosis-factor receptor family. Gene.

[18] Alderson Mark R., Smith Craig A., Tough Teresa W., Davis-Smith T., Armitage Richard J., Falk B., Roux E., Baker E., Sutherland Grant R., Din Wenie S. (1994). Moslecular and biological characterization of human 4-1BB and its ligands. Eur. J. Immunol..

[19] Vinay D.S., Kwon B.S. (2016). Therapeutic potential of anti-CD137 (4-1BB) monoclonal antibodies. Expert Opin. Ther. Targets.

[20] Cannons J.L., Choi Y., Watts T.H. (2000). Role of TNF Receptor-Associated Factor 2 and p38 Mitogen-Activated Protein Kinase Activation During 4-1BB-Dependent Immune Response. J. Immunol..

[21] Takahashi C., Mittler R.S., Vella A.T. (1999). Cutting Edge: 4-1BB Is a Bona Fide CD8 T Cell Survival Signal. J. Immunol..

[22] Lee H.-W., Park S.-J., Choi B.K., Kim H.H., Nam K.-O., Kwon B.S. (2002). 4-1BB promotes the survival of CD8+ T lymphocytes by increasing expression of Bcl-xL and Bfl-1. J. Immunol..

[23] Cole S.L., Benam K.H., McMichael A.J., Ho L.-P. (2014). Involvement of the 4-1BB/4-1BBL Pathway in Control of Monocyte Numbers by Invariant NKT Cells. J. Immunol..

[24] Bostrom P., Wu J., Jedrychowski M.P., Korde A., Ye L., Lo J.C., Rasbach K.A., Bostrom E.A., Choi J.H., Long J.Z. (2012). A PGC1-α-dependent myokine that drives brown-fat-like development of white fat and thermogenesis. Nature.

[25] Pollok Karen E., Kim Y.-J., Hurtado J., Zhou Z., Kim Kack K., Kwon Byoung S. (1994). 4-1BB T-cell antigen binds to mature B cells and macrophages, and costimulates anti-μ-primed splenic B cells. Eur. J. Immunol..

[26] Gauttier V., Judor J.-P., Le Guen V., Cany J., Ferry N., Conchon S. (2014). Agonistic anti-CD137 antibody treatment leads to antitumor response in mice with liver cancer. Int. J. Cancer.

[27] Narazaki H., Zhu Y., Luo L., Zhu G., Chen L. (2010). CD137 agonist antibody prevents cancer recurrence: Contribution of CD137 on both hematopoietic and nonhematopoietic cells. Blood.

[28] Houot R., Goldstein M.J., Kohrt H.E., Myklebust J.H., Alizadeh A.A., Lin J.T., Irish J.M., Torchia J.A., Kolstad A., Chen L. (2009). Therapeutic effect of CD137 immunomodulation in lymphoma and its enhancement by Treg depletion. Blood.

[29] Palazón A., Teijeira A., Martínez-Forero I., Hervás-Stubbs S., Roncal C., Peñuelas I., Dubrot J., Morales-Kastresana A., Pérez-Gracia J.L., Ochoa M.C. (2011). Agonist Anti-CD137 mAb Act on Tumor Endothelial Cells to Enhance Recruitment of Activated T Lymphocytes. Cancer Res..

[30] Morales-Kastresana A., Catalan E., Hervas-Stubbs S., Palazon A., Azpilikueta A., Bolanos E., Anel A., Pardo J., Melero I. (2013). Essential complicity of perforin-granzyme and FAS-L mechanisms to achieve tumor rejection following treatment with anti-CD137 mAb. J. Immunother. Cancer.

[31] Shi W., Siemann D.W. (2006). Augmented Antitumor Effects of Radiation Therapy by 4-1BB Antibody (BMS-469492) Treatment. Anticancer Res..

[32] Chacon J.A., Wu R.C., Sukhumalchandra P., Molldrem J.J., Sarnaik A., Pilon-Thomas S., Weber J., Hwu P., Radvanyi L. (2013). Co-Stimulation through 4-1BB/CD137 Improves the Expansion and Function of CD8^+^ Melanoma Tumor-Infiltrating Lymphocytes for Adoptive T-Cell Therapy. PLoS ONE.

[33] Melero I., Hirschhorn-Cymerman D., Morales-Kastresana A., Sanmamed M.F., Wolchok J.D. (2013). Agonist Antibodies to TNFR Molecules That Costimulate T and NK Cells. Clin. Cancer Res..

[34] Melero I., Grimaldi A.M., Perez-Gracia J.L., Ascierto P.A. (2013). Clinical Development of Immunostimulatory Monoclonal Antibodies and Opportunities for Combination. Clin. Cancer Res..

[35] Madireddi S., Eun S.-Y., Lee S.-W., Nemčovičová I., Mehta A.K., Zajonc D.M., Nishi N., Niki T., Hirashima M., Croft M. (2014). Galectin-9 controls the therapeutic activity of 4-1BB–targeting antibodies. J. Exp. Med..

[36] Palazón A., Martínez-Forero I., Teijeira A., Morales-Kastresana A., Alfaro C., Sanmamed M.F., Perez-Gracia J.L., Peñuelas I., Hervás-Stubbs S., Rouzaut A. (2012). The HIF-1α Hypoxia Response in Tumor-Infiltrating T Lymphocytes Induces Functional CD137 (4-1BB) for Immunotherapy. Cancer Discov..

[37] Martinez-Forero I., Azpilikueta A., Bolaños-Mateo E., Nistal-Villan E., Palazon A., Teijeira A., Perez-Chacon G., Morales-Kastresana A., Murillo O., Jure-Kunkel M. (2013). T Cell Costimulation with Anti-CD137 Monoclonal Antibodies Is Mediated by K63–Polyubiquitin-Dependent Signals from Endosomes. J. Immunol..

[38] Curran M.A., Kim M., Montalvo W., Al-Shamkhani A., Allison J.P. (2011). Combination CTLA-4 Blockade and 4-1BB Activation Enhances Tumor Rejection by Increasing T-Cell Infiltration, Proliferation, and Cytokine Production. PLoS ONE.

[39] James A.M., Cohen A.D., Campbell K.S. (2013). Combination immune therapies to enhance anti-tumor responses by NK cells. Front. Immunol..

[40] Smith S., Hoelzinger D., Dominguez A., Van Snick J., Lustgarten J. (2011). Signals through 4-1BB inhibit T regulatory cells by blocking IL-9 production enhancing antitumor responses. Cancer Immunol. Immunother..

[41] Fisher T., Kamperschroer C., Oliphant T., Love V., Lira P., Doyonnas R., Bergqvist S., Baxi S., Rohner A., Shen A. (2012). Targeting of 4-1BB by monoclonal antibody PF-05082566 enhances T-cell function and promotes anti-tumor activity. Cancer Immunol. Immunother..

[42] Yi L., Zhao Y., Wang X., Dai M., Hellström K.E., Hellström I., Zhang H. (2014). Human and Mouse CD137 Have Predominantly Different Binding CRDs to Their Respective Ligands. PLoS ONE.

[43] Snell L.M., Lin G.H.Y., McPherson A.J., Moraes T.J., Watts T.H. (2011). T-cell intrinsic effects of GITR and 4-1BB during viral infection and cancer immunotherapy. Immunol. Rev..

[44] Chester C., Ambulkar S., Kohrt H.E. (2016). 4-1BB agonism: Adding the accelerator to cancer immunotherapy. Cancer Immunol. Immunother..

[45] Sznol M., Hodi F.S., Margolin K., McDermott D.F., Ernstoff M.S., Kirkwood J.M., Wojtaszek C., Feltquate D., Logan T. (2008). Phase I study of BMS-663513, a fully human anti-CD137 agonist monoclonal antibody, in patients (pts) with advanced cancer (CA). J. Clin. Oncol..

[46] Segal N.H., Logan T.F., Hodi F.S., McDermott D., Melero I., Hamid O., Schmidt H., Robert C., Chiarion-Sileni V., Ascierto P.A. (2017). Results from an Integrated Safety Analysis of Urelumab, an Agonist Anti-CD137 Monoclonal Antibody. Clin. Cancer Res..

[47] Zhang J., Song K., Wang J., Li Y., Liu S., Dai C., Chen L., Wang S., Qin Z. (2018). S100A4 blockage alleviates agonistic anti-CD137 antibody-induced liver pathology without disruption of antitumor immunity. OncoImmunology.

[48] Massarelli E. Clinical safety and efficacy assessment of the CD137 agonist urelumab alone and in combination with nivolumab in patients with hematologic and solid tumor malignancies. Proceedings of the 31st Annual Meeting & Associated Programs of the Society for Immunotherapy of Cancer (SITC)’s.

[49] Segal N.H., Gopal A.K., Bhatia S., Kohrt H.E., Levy R., Pishvaian M.J., Houot R., Bartlett N., Nghiem P., Kronenberg S.A. (2014). A phase 1 study of PF-05082566 (anti-4-1BB) in patients with advanced cancer. J. Clin. Oncol..

[50] Segal N.H., He A.R., Doi T., Levy R., Bhatia S., Pishvaian M.J., Cesari R., Chen Y., Davis C.B., Huang B. (2018). Phase I Study of Single-Agent Utomilumab (PF-05082566), a 4-1BB/CD137 Agonist, in Patients with Advanced Cancer. Clin. Cancer Res..

[51] Chin S.M., Kimberlin C.R., Roe-Zurz Z., Zhang P., Xu A., Liao-Chan S., Sen D., Nager A.R., Oakdale N.S., Brown C. (2018). Structure of the 4-1BB/4-1BBL complex and distinct binding and functional properties of utomilumab and urelumab. Nat. Commun..

[52] Chester C., Sanmamed M.F., Wang J., Melero I. (2018). Immunotherapy targeting 4-1BB: Mechanistic rationale, clinical results, and future strategies. Blood.

[53] Kerage D., Soon M.S.F., Doff B.L., Kobayashi T., Nissen M.D., Lam P.Y., Leggatt G.R., Mattarollo S.R. (2018). Therapeutic vaccination with 4–1BB co-stimulation eradicates mouse acute myeloid leukemia. OncoImmunology.

[54] Murillo O., Arina A., Hervas-Stubbs S., Gupta A., McCluskey B., Dubrot J., Palazón A., Azpilikueta A., Ochoa M.C., Alfaro C. (2008). Therapeutic Antitumor Efficacy of Anti-CD137 Agonistic Monoclonal Antibody in Mouse Models of Myeloma. Clin. Cancer Res..

[55] Wei H., Zhao L., Li W., Fan K., Qian W., Hou S., Wang H., Dai M., Hellstrom I., Hellstrom K.E. (2013). Combinatorial PD-1 Blockade and CD137 Activation Has Therapeutic Efficacy in Murine Cancer Models and Synergizes with Cisplatin. PLoS ONE.

[56] Choi B.K., Kim Y.H., Kang W.J., Lee S.K., Kim K.H., Shin S.M., Yokoyama W.M., Kim T.Y., Kwon B.S. (2007). Mechanisms Involved in Synergistic Anticancer Immunity of Anti-4-1BB and Anti-CD4 Therapy. Cancer Res..

[57] Westwood J.A., Matthews G.M., Shortt J., Faulkner D., Pegram H.J., Duong C.P.M., Chesi M., Bergsagel P.L., Sharp L.L., Huhn R.D. (2014). Combination anti-CD137 and anti-CD40 antibody therapy in murine myc-driven hematological cancers. Leuk. Res..

[58] Kocak E., Lute K., Chang X., May K.F., Exten K.R., Zhang H., Abdessalam S.F., Lehman A.M., Jarjoura D., Zheng P. (2006). Combination Therapy with Anti–CTL Antigen-4 and Anti-4-1BB Antibodies Enhances Cancer Immunity and Reduces Autoimmunity. Cancer Res..

[59] Simeone E., Ascierto P.A. (2012). Immunomodulating antibodies in the treatment of metastatic melanoma: The experience with anti-CTLA-4, anti-CD137, and anti-PD1. J. Immunotoxicol..

[60] Dai M., Wei H., Yip Y.Y., Feng Q., He K., Popov V., Hellstrom I., Hellstrom K.E. (2013). Long-lasting Complete Regression of Established Mouse Tumors by Counteracting Th2 Inflammation. J. Immunother..

[61] Ascierto P.A., Kalos M., Schaer D.A., Callahan M.K., Wolchok J.D. (2013). Biomarkers for Immunostimulatory Monoclonal Antibodies in Combination Strategies for Melanoma and Other Tumor Types. Clin. Cancer Res..

[62] Ascierto P., Capone M., Urba W., Bifulco C., Botti G., Lugli A., Marincola F., Ciliberto G., Galon J., Fox B. (2013). The additional facet of immunoscore: Immunoprofiling as a possible predictive tool for cancer treatment. J. Transl. Med..

[63] Morales-Kastresana A., Sanmamed M.F., Rodriguez I., Palazon A., Martinez-Forero I., Labiano S., Hervas-Stubbs S., Sangro B., Ochoa C., Rouzaut A. (2013). Combined Immunostimulatory Monoclonal Antibodies Extend Survival in an Aggressive Transgenic Hepatocellular Carcinoma Mouse Model. Clin. Cancer Res..

[64] Guo Z., Cheng D., Xia Z., Luan M., Wu L., Wang G., Zhang S. (2013). Combined TIM-3 blockade and CD137 activation affords the long-term protection in a murine model of ovarian cancer. J. Transl. Med..

[65] Lee C., Tannock I. (2010). The distribution of the therapeutic monoclonal antibodies cetuximab and trastuzumab within solid tumors. BMC Cancer.

[66] Kohrt H.E., Colevas A.D., Houot R., Weiskopf K., Goldstein M.J., Lund P., Mueller A., Sagiv-Barfi I., Marabelle A., Lira R. (2014). Targeting CD137 enhances the efficacy of cetuximab. J. Clin. Investig..

[67] Chen L., Li J., Zhang J., Dai C., Liu X., Wang J., Gao Z., Guo H., Wang R., Lu S. (2015). S100A4 promotes liver fibrosis via activation of hepatic stellate cells. J. Hepatol..

[68] Hansen M.T., Forst B., Cremers N., Quagliata L., Ambartsumian N., Grum-Schwensen B., Klingelhöfer J., Abdul-Al A., Herrmann P., Osterland M. (2014). A link between inflammation and metastasis: Serum amyloid A1 and A3 induce metastasis, and are targets of metastasis-inducing S100A4. Oncogene.

[69] Schmidt-Hansen B., Örnås D., Grigorian M., Klingelhöfer J., Tulchinsky E., Lukanidin E., Ambartsumian N. (2004). Extracellular S100A4(mts1) stimulates invasive growth of mouse endothelial cells and modulates MMP-13 matrix metalloproteinase activity. Oncogene.

[70] Yonezawa A., Dutt S., Chester C., Kim J., Kohrt H.E. (2015). Boosting Cancer Immunotherapy with Anti-CD137 Antibody Therapy. Clin. Cancer Res..

[71] Hosoi A., Takeda K., Nagaoka K., Iino T., Matsushita H., Ueha S., Aoki S., Matsushima K., Kubo M., Morikawa T. (2018). Increased diversity with reduced “diversity evenness” of tumor infiltrating T-cells for the successful cancer immunotherapy. Sci. Rep..

[72] McKee S.J., Doff B.L., Soon M.S.F., Mattarollo S.R. (2017). Therapeutic Efficacy of 4-1BB Costimulation Is Abrogated by PD-1 Blockade in a Model of Spontaneous B-cell Lymphoma. Cancer Immunol. Res..

[73] Verbrugge I., Hagekyriakou J., Sharp L.L., Galli M., West A., McLaughlin N.M., Duret H., Yagita H., Johnstone R.W., Smyth M.J. (2012). Radiotherapy Increases the Permissiveness of Established Mammary Tumors to Rejection by Immunomodulatory Antibodies. Cancer Res..

[74] Jensen B.A.H., Pedersen S.R., Christensen J.P., Thomsen A.R. (2013). The Availability of a Functional Tumor Targeting T-Cell Repertoire Determines the Anti-Tumor Efficiency of Combination Therapy with Anti-CTLA-4 and Anti-4-1BB Antibodies. PLoS ONE.

[75] Verbrugge I., Gasparini A., Haynes N.M., Hagekyriakou J., Galli M., Stewart T.J., Abrams S.I., Yagita H., Verheij M., Johnstone R.W. (2014). The Curative Outcome of Radioimmunotherapy in a Mouse Breast Cancer Model Relies on mTOR Signaling. Radiat. Res..

[76] Hebb J.P.O., Mosley A.R., Vences-Catalán F., Rajasekaran N., Rosén A., Ellmark P., Felsher D.W. (2018). Administration of low-dose combination anti-CTLA4, anti-CD137, and anti-OX40 into murine tumor or proximal to the tumor draining lymph node induces systemic tumor regression. Cancer Immunol. Immunother..

[77] Kohrt H.E., Houot R., Weiskopf K., Goldstein M.J., Scheeren F., Czerwinski D., Colevas A.D., Weng W.-K., Clarke M.F., Carlson R.W. (2012). Stimulation of natural killer cells with a CD137-specific antibody enhances trastuzumab efficacy in xenotransplant models of breast cancer. J. Clin. Investig..

[78] Stagg J., Loi S., Divisekera U., Ngiow S.F., Duret H., Yagita H., Teng M.W., Smyth M.J. (2011). Anti–ErbB-2 mAb therapy requires type I and II interferons and synergizes with anti–PD-1 or anti-CD137 mAb therapy. Proc. Natl. Acad. Sci. USA.

[79] Tolcher A.W., Sznol M., Hu-Lieskovan S., Papadopoulos K.P., Patnaik A., Rasco D.W., Di Gravio D., Huang B., Gambhire D., Chen Y. (2017). Phase Ib Study of Utomilumab (PF-05082566), a 4-1BB/CD137 Agonist, in Combination with Pembrolizumab (MK-3475) in Patients with Advanced Solid Tumors. Clin. Cancer Res..

[80] Xu D., Gu P., Pan P.-Y., Li Q., Sato A.I., Chen S.-H. (2004). NK and CD8+ T cell-mediated eradication of poorly immunogenic B16-F10 melanoma by the combined action of IL-12 gene therapy and 4-1BB costimulation. Int. J. Cancer.

[81] Tirapu I., Arina A., Mazzolini G., Duarte M., Alfaro C., Feijoo E., Qian C., Chen L., Prieto J., Melero I. (2004). Improving efficacy of interleukin-12-transfected dendritic cells injected into murine colon cancer with anti-CD137 monoclonal antibodies and alloantigens. Int. J. Cancer.

[82] Ito F., Li Q., Shreiner A.B., Okuyama R., Jure-Kunkel M.N., Teitz-Tennenbaum S., Chang A.E. (2004). Anti-CD137 Monoclonal Antibody Administration Augments the Antitumor Efficacy of Dendritic Cell-Based Vaccines. Cancer Res..

[83] Quetglas J.I., Dubrot J., Bezunartea J., Sanmamed M.F., Hervas-Stubbs S., Smerdou C., Melero I. (2012). Immunotherapeutic Synergy Between Anti-CD137 mAb and Intratumoral Administration of a Cytopathic Semliki Forest Virus Encoding IL-12. Mol. Ther..

[84] Westwood J.A., Potdevin Hunnam T.C.U., Pegram H.J., Hicks R.J., Darcy P.K., Kershaw M.H. (2014). Routes of Delivery for CpG and Anti-CD137 for the Treatment of Orthotopic Kidney Tumors in Mice. PLoS ONE.

[85] Ko E., Luo W., Peng L., Wang X., Ferrone S. (2007). Mouse Dendritic-Endothelial Cell Hybrids and 4-1BB Costimulation Elicit Antitumor Effects Mediated by Broad Antiangiogenic Immunity. Cancer Res..

[86] Chen S.-H., Pham-Nguyen K.B., Martinet O., Huang Y., Yang W., Thung S.N., Chen L., Mittler R., Woo S.L. (2000). Rejection of Disseminated Metastases of Colon Carcinoma by Synergism of IL-12 Gene Therapy and 4-1BB Costimulation. Mol. Ther..

[87] Li B., Lin J., VanRoey M., Jure-Kunkel M., Jooss K. (2007). Established B16 tumors are rejected following treatment with GM-CSF-secreting tumor cell immunotherapy in combination with anti-4-1BB mAb. Clin. Immunol..

[88] Ju S.-A., Cheon S.-H., Park S.-M., Tam N.Q., Kim Y.M., An W.G., Kim B.-S. (2008). Eradication of established renal cell carcinoma by a combination of 5-fluorouracil and anti-4-1BB monoclonal antibody in mice. Int. J. Cancer.

[89] John L.B., Howland L.J., Flynn J.K., West A.C., Devaud C., Duong C.P., Stewart T.J., Westwood J.A., Guo Z.S., Bartlett D.L. (2012). Oncolytic Virus and Anti–4-1BB Combination Therapy Elicits Strong Antitumor Immunity against Established Cancer. Cancer Res..

[90] Park J.-J., Anand S., Zhao Y., Matsumura Y., Sakoda Y., Kuramasu A., Strome S., Chen L., Tamada K. (2012). Expression of anti-HVEM single-chain antibody on tumor cells induces tumor-specific immunity with long-term memory. Cancer Immunol. Immunother..

[91] Kobayashi T., Doff B.L., Rearden R.C., Leggatt G.R., Mattarollo S.R. (2015). NKT cell-targeted vaccination plus anti-4–1BB antibody generates persistent CD8 T cell immunity against B cell lymphoma. OncoImmunology.

[92] Wilcox R.A., Tamada K., Strome S.E., Chen L. (2002). Signaling Through NK Cell-Associated CD137 Promotes Both Helper Function for CD8+ Cytolytic T Cells and Responsiveness to IL-2 But Not Cytolytic Activity. J. Immunol..

[93] Kohrt H.E., Houot R., Goldstein M.J., Weiskopf K., Alizadeh A.A., Brody J., Müller A.M.S., Pachynski R., Czerwinski D., Coutre S. (2010). CD137 stimulation enhances the anti-lymphoma activity of anti-CD20 antibodies. Blood.

[94] Knight D.A., Ngiow S.F., Li M., Parmenter T., Mok S., Cass A., Haynes N.M., Kinross K., Yagita H., Koya R.C. (2013). Host immunity contributes to the anti-melanoma activity of BRAF inhibitors. J. Clin. Investig..

[95] Kwong B., Gai S.A., Elkhader J., Wittrup K.D., Irvine D.J. (2013). Localized Immunotherapy via Liposome-Anchored Anti-CD137 + IL-2 Prevents Lethal Toxicity and Elicits Local and Systemic Antitumor Immunity. Cancer Res..

[96] Zhang Y., Li N., Suh H., Irvine D.J. (2018). Nanoparticle anchoring targets immune agonists to tumors enabling anti-cancer immunity without systemic toxicity. Nat. Commun..

[97] Ribas A., Wolchok J.D. (2018). Cancer immunotherapy using checkpoint blockade. Science.

[98] Ledford H., Else H., Warren M. (2018). Cancer immunologists scoop medicine Nobel prize. Nature.

[99] Pilones K., Aryankalayil J., Babb J., Demaria S. (2014). Invariant natural killer T cells regulate anti-tumor immunity by controlling the population of dendritic cells in tumor and draining lymph nodes. J. Immunother. Cancer.

